# Obesity‐Induced Loss of Function of Bone Marrow Mesenchymal Stromal Cells Is Linked to Cellular Stress and Irreversible at Advanced Stages

**DOI:** 10.1111/jcmm.70776

**Published:** 2025-08-07

**Authors:** Ece Gizem Polat, Mehmet Emin Şeker, Burcu Pervin, Barış Ulum, Fatima Aerts‐Kaya

**Affiliations:** ^1^ Department of Stem Cell Sciences Hacettepe University Graduate School of Health Sciences Ankara Turkey; ^2^ Hacettepe University Center for Stem Cell Research and Development Ankara Turkey; ^3^ Pediatric Basic Sciences, Department of Pediatric Immunology Hacettepe University Institute of Child Health Ankara Turkey

**Keywords:** bone marrow, endoplasmic reticulum stress, obesity, oxidative stress, senescence

## Abstract

Obesity increases the likelihood of metabolic diseases and can affect stem cell function negatively. Here, we aimed to elucidate the mechanisms involved in the loss of stem cell function induced by obesity by assessing levels of oxidative stress (OS) and endoplasmic reticulum stress (ERS) in bone marrow‐derived mesenchymal stromal cells (BM‐MSCs) from healthy donors with a body mass index (BMI) of 25–30 (obese) and BMI > 30 (morbid obese). We assessed base levels of OS and ERS, activation of cellular response mechanisms, and the effects of Melatonin (MT), which is known to decrease OS, and TUDCA, which is known to decrease ERS. Loss of BM‐MSC differentiation was correlated with the degree of obesity and associated with upregulation of OS and ERS. Increased BMI was accompanied by elevated intracellular ROS and accelerated senescence of BM‐MSCs. Although treatment with MT and TUDCA was able to decrease OS and ERS in BM‐MSCs from obese donors, cellular stress in BM‐MSCs from morbid obese donors was irreversible. Therefore, it is imperative to treat and prevent obesity before the negative effects on stem cells become permanent and irreversible. Early treatment of obesity may not only prevent metabolic diseases; it may also protect tissue resident stem cells.

## 1 Introduction

Obesity has become a global public health problem and increases the likelihood of metabolic diseases, such as diabetes and cardiovascular diseases [1]. Obesity is quantified in adults using the body mass index (BMI), where a BMI of 20–25 represents healthy individuals, BMI ≥ 25 represents obese individuals, and BMI ≥ 30 represents morbidly obese patients. The field of use of stem cells for regenerative medicine purposes has been rapidly expanding, and recent studies indicate that obesity affects stem cell function [2, 3]. Due to the elevated rates of obesity in the general population, it has become increasingly important to elucidate the potential (negative) effects of obesity on stem cell function for two reasons: firstly, if stem cell and tissue repair functions are affected by obesity, this could be used as a treatment target; secondly, the autologous use of stem cells for regenerative medicine is rapidly increasing, and infusion or use of functionally impaired autologous stem cells may not result in optimal regenerative/immune modulatory effects. Indeed, it has been shown that obesity causes functional impairment of bone marrow (BM)‐derived mesenchymal stromal/stem cells (MSCs) from humans and mice and reduces their regenerative potential [2, 3]. BM‐MSCs obtained from human donors with an increased BMI exhibit severely impaired osteogenic and reduced adipogenic differentiation, reduced proliferation rates, and increased expression of the endoplasmic reticulum (ER) stress‐associated genes *XBP1*, *ATF4*, and *CHOP* [2]. Similarly, the differentiation and proliferative potential of adipose tissue‐derived stem cells (ADSCs) from obese individuals are impaired [4]. In addition, ADSCs from obese individuals also showed an increased senescent cell burden and phenotype, as apparent by p16, p53, *IL‐6*, and *MCP‐1* gene expression, that correlated directly with BMI [5]. Obesity‐induced cellular injury therefore limits the use of ADSCs for endogenous repair systems or autologous use in obese individuals [5, 6]. Obesity also negatively affects the functions of many other stem cell types, including skeletal muscle stem cells and germ cells [4, 7, 8, 9] and an obesity‐related increase in BM adipocytes may indirectly cause haematopoietic stem/progenitor cell dysfunction and immune deficiency [10].

BM‐MSCs are multipotent cells with high self‐renewal abilities that can differentiate into chondrocytes, osteocytes, and adipocytes [11, 12]. Due to these characteristics, as well as their low immunogenicity [13], immunomodulatory properties [14], easy collection and expansion, and their ability to home to sites of injury [15], MSCs have become especially important in the field of regenerative medicine and in the treatment of (auto)immune diseases [16]. However, cellular stress, such as ER stress (ERS), caused by an increase in misfolded/unfolded proteins and oxidative stress (OS), induced by an increase in reactive oxygen species (ROS) has been shown to affect stem cells negatively [17, 18]. When the cellular defence mechanisms that protect the cells from ERS or OS fail, cellular stress can become chronic, causing the onset and progression of various human diseases, including neurodegenerative and metabolic diseases [19, 20].

Therefore, addressing or targeting cellular stress in stem cells from high BMI individuals may prove to be beneficial to preserve or regain stem cell function. Methods to do so include the use of antioxidant molecules to reduce OS and protect the cell from prolonged stress, by direct scavenging of ROS or upregulation of antioxidant genes, including superoxide dismutase 1 (*SOD1*) and thioredoxin reductase 1 (*TRXNRD1*) [21, 22]. Alternatively, drugs to decrease ERS and suppress the unfolded protein response (UPR) that is activated in response to increased levels of unfolded/misfolded proteins, such as Tauroursodeoxycholic acid (TUDCA) [2, 19] can be used together with antioxidants, such as Melatonin (MT) [23]. Indeed, MT has an important role in the regulation of antioxidant defence mechanisms, modulation of the immune system, and cancer prevention [24, 25]. In addition, metabolites of MT have also been shown to harbour an antioxidant function [26]. Other mechanisms through which MT may exert its effects are through the binding of metals, reducing ^o^OH radicals, and activation of antioxidant enzyme systems, such as SOD, glutathion peroxidase (GPx) and glutathion reductase [23].

Here, we aimed to assess the relationship between increasing BMI and BM‐MSC function by assessing baseline levels and induction of ERS markers, as well as by assessing indicators of OS during culture and differentiation of BM‐MSCs derived from healthy donors (BMI 20–25), obese donors (BMI 25–30) and morbid obese donors (BMI > 30), before and after treatment with TUDCA and/or MT.

## 2 Materials and Methods

### 2.1 Isolation and Characterisation of BM‐MSCs

BM‐MSCs were isolated from physically healthy BM donors at the Paediatric Haematology Bone Marrow Transplantation Unit after approval of Hacettepe University Ethics Committee (GO:2022/11–68). BM was collected from adult (age 18 years) healthy (BMI 20–25, *n* = 3), obese (BMI 25–30, *n* = 3) and morbid obese (BMI > 30, *n* = 3) donors. Exclusion criteria included the presence of diabetes, hypertension or co‐morbidities, such as cancer. Cholesterol levels, liver or kidney functions of the donors were not assessed. The mononuclear cells were cultured in a basal medium mixture consisting of 40% DMEM‐LG (Gibco, 31885‐049)/60% MCDB‐201 (Sigma, M6770‐1L), supplemented with 10% Fetal Calf Serum [FBS] (Gibco, 10270‐106), 1% Penicillin/Streptomycin (Biochrom, A2213), 2 mM L‐Glutamine (Biochrom AG), or briefly DMF10. Cells were cultured at 37°C, 5% CO_2_ and the medium of the cells was changed every 3–4 days. For immunophenotyping, BM‐MSC/P3 cells were collected using 0.25% Trypsin/EDTA and stained with directly labelled antibodies against CD29, CD73, CD105, CD90, HLA‐DR and CD45 (all purchased from BioLegend) in PBS with BSA and NaN_3_ (PBN) with 2% AB serum.

For adipogenic differentiation, passage 3 (P3) BM‐MSCs were differentiated in the presence of adipogenic differentiation medium, consisting of DMEM‐LG, 10% FBS, 1 μM dexamethasone, 60 μM indomethacin, 500 μM isobutylmethylxanthine, and 5 μg/mL insulin. Differentiation was evaluated using RT‐qPCR to assess the expression of the adipogenic differentiation markers *PPARG* and *SCD*. The presence of lipids was evaluated with Oil Red O (ORO) staining, as described before [2]. For osteogenic differentiation, BM‐MSCs were differentiated in DMEM‐LG, 10% FBS, 100 nM dexamethasone, 10 mM betaglycerophosphate, and 0.2 mM L‐ascorbic acid. Osteogenic differentiation was evaluated using RT‐qPCR for gene expression of the osteogenic differentiation markers *ALPL* and *RUNX2*. Cells were further fixed and stained with Alizarin Red S at pH 4.2. Calcium content was assessed using the Quantichrom Calcium assay kit (BioAssay systems, DICA500), as described before [2]. For chondrogenic differentiation, 2 × 10^5^ cells were transferred to 15 mL polypropylene tubes. Cell pellets were left overnight at 37°C, 5% CO_2_, and allowed to form small spheres. Chondrocyte differentiation medium, consisting of DMEM‐HG (Gibco, 20958‐22), 1 mM Sodium Pyruvate (Sigma, SML0309), 100 nM dexamethasone, 50 μg/mL L‐Ascorbic acid, and 10 g/mL TGF‐β3 (Immunotools), 1X ITS (BD Biosciences, I‐1884) was used to induce differentiation. After differentiation, cell spheres were washed and used for RT‐qPCR. Chondrogenic differentiation was assessed using the cartilage‐specific gene markers *SOX9* and *COL2*.

### 2.2 Evaluation of Cellular Stress and Senescence in BM‐MSCs

To investigate the effects of obesity on OS in BM‐MSCs, intracellular ROS levels were evaluated in BM‐MSCs from healthy, obese, and morbid obese donors using H2DCFDA (2′,7′‐dichlorodihydrofluorescein‐diacetate) (Thermofisher Scientific, D399), according to the manufacturer's instructions. Briefly, passage 6 (P6) BM‐MSCs were used for analysis. Negative control BM‐MSCs (unstained) and BM‐MSCs from BMI 20–25, BMI 25–30, and BMI > 30 donors were incubated in DMF10 only (without any induction) to assess baseline levels of OS. Positive controls were incubated in DMF10 with 100 μM H_2_O_2_ (Sigma, H1009) for 2 h at 37°C, 5% CO_2_, and were used for gating purposes. Samples were incubated with 10 μM H2DCFDA in pre‐warmed PBS for 30 min. Cells were washed three times with ice‐cold PBS, and intracellular ROS levels were evaluated on a BDAccuri flow cytometer (Becton Dickinson).

The expression of the OS‐related genes, *SOD1* and *TRXNRD1* was assessed in BM‐MSCs from healthy, obese and morbid obese donors using RT‐qPCR. To assess ERS, gene expression of *sXBP1*, *XBP1*, *ATF4* and *CHOP* was examined. BM‐MSCs/P6 were collected in Qiazol (Qiagen, 57201570). RNA was isolated using a total RNA isolation kit (Zymo, R2062). Forward and reverse sequences of primers used are given in Table S1. mRNAs were converted into cDNA using the Quantitect Reverse Transcription Kit (Applied Biosystems, 00314158). Gene expression was analysed using a LightCycler 480‐II (Roche). *RPLP0* gene expression was used as a house keeping gene, in line with previous data [27]. All experiments were performed in triplicate.

Differences in the expression of senescence‐associated β‐galactosidase (SA‐β‐Gal) in healthy, obese, and morbidly obese BM‐MSC/P6 cells were measured using the SA‐β‐Gal kit (Cayman Chemical Company). BM‐MSCs/P6 were fixed in fixation solution for 15 min at room temperature, washed twice with PBS, and incubated with 1 mL of β‐Gal staining solution at pH 5.9–6.1 overnight at 37°C. Images were taken using an inverted light microscope, showing blue (SA‐β‐Gal positive) stain as an indicator for senescent cells and evaluated with ImageJ software, as previously described [2]. In order to be able to distinguish between endogenous galactosidase expression and ‘true’ senescence, wells stained with SA‐β‐Gal were subsequently permeabilised with 0.3% Triton X‐100 in PBS, followed by blocking with 5% FBS and 0.1% Triton X‐100 in PBS for 1 h at RT and staining with 1:500 diluted rabbit‐anti‐human Ki67 (Thermofisher Scientific, MA5‐14520) overnight. The next day, wells were stained with 1:10,000 donkey‐anti‐rabbit‐IgG‐FITC (Thermofisher Scientific, A16024). Wells were counterstained with 5 μg/mL 4′,6‐Diamidino‐2‐phenylindole (DAPI, Abcam, ab285390) for 5 min. Pictures were taken using an Olympus IX‐73 fluorescent microscope and assessed using free online ImageJ software. Senescent cells were identified as Ki67‐/SA‐β‐gal+ cells [28].

### 2.3 Dose Determination and Assessment of Downstream Signalling of MT in Healthy BM‐MSCs

To assess possible negative effects of MT, healthy donor BM‐MSCs were exposed to 0–300 nM of MT for 24 h. Then, cells were collected in Annexin binding buffer and stained with Annexin‐V‐FITC (Ann‐V) and Propidium Iodide (PI), according to the manufacturer's instructions (Biolegend, 640,906).

To test the effect of MT on cell proliferation, healthy donor BM‐MSCs were cultured and cell expansion was assessed using WST‐1 (Roche, 11644807001). Cells were plated into 96‐well plates in triplicate at 2000 cells/well and cultured in DMF10 with or without 50 μM TUDCA and/or 30 nM MT. WST‐1 reagent was added to wells at days 4, 7, 11, and 14 after culture at a dose of 1:10. Cells were then incubated for 4 h at 37°C, 5% CO_2_, and absorbance was measured with a microplate reader (Tecan Sunrise) at 420–480 nm.

To assess downstream signalling of BM‐MSCs in response to MT, healthy donor BM‐MSCs were starved overnight and 24 h later exposed to 30 nM MT at 37°C, 5% CO_2_. Cells were collected before (0′) and 15′, 30′, 45′, 60′, and 90′ min after application of MT. Directly after collection, cells were fixed in 500 μL Fix Buffer 1 (BD Bioscience, 557870) at 37°C for 10 min. The cells were washed twice with 2 mL PBN and resuspended in 500 μL of ice‐cold PBN for 30 min. For staining of phosphoproteins, cells were incubated with anti‐pERK‐Alexa Fluor 488 (Biolegend, 675508) and 10 μL of anti‐pAkt‐PE (BD Bioscience, 560378) antibodies in 100 μL of PBN + AB Serum for 15 min at room temperature. Cells were washed twice with 2 mL PBN, and assessed using the BDAccuri.

### 2.4 Statistical Analysis

Significancy of differences was calculated with the Student's *T*‐test using Excel for Mac version 14.6.9 software. A *p* value of < 0.05 was considered significant. Fold changes in gene expression during differentiation of BM‐MSCs and before and after treatment with TUDCA and MT were calculated using the relative ($\Delta \Delta C_{t}$ method) [27]. Correlations were calculated with the Pearson's test.

## 3 Results

### 3.1 BM‐MSCs From Healthy and Obese Donors Are Morphologically and Phenotypically Similar

**FIGURE 1:**
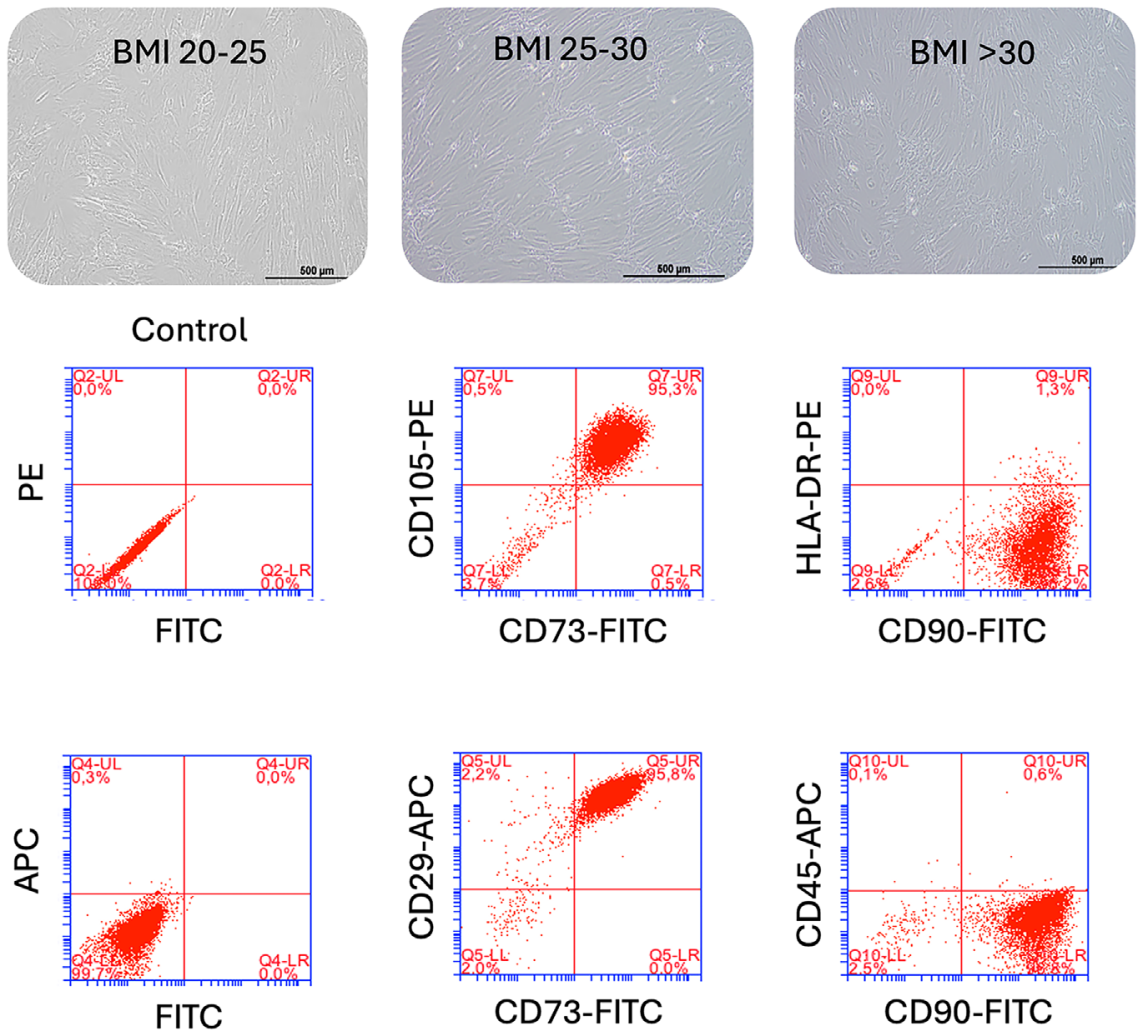
Morphology and immunophenotype of healthy and obese donor BM‐MSCs. Upper panel: BM‐MSCs were obtained from healthy (BMI 20–25, left), obese (BMI 25–30, middle) and morbid obese donors (BMI > 30, right). Morphologically cells highly resembled each other. Lower panel: Flow cytometry dot plots from a representative healthy donor BM‐MSC sample.

Clinically relevant data of the BM donors used in this study are provided in Table 1. Groups were stratified according to BMI, with a BMI of 20–25 representing healthy, BMI 25–30 obese, and a BMI > 30 morbid obese donors. Although BMI was significantly different between the groups (*p* < 0.05), differences in donor age were not significant (*p* > 0.2). Since there was also no positive correlation between age and BMI in the selected donors (*r* = 0.35, *p* > 0.05), we concluded that age is not a confounding factor or variable in this study. Morphologically, BM‐MSCs from healthy and obese donors appeared similar. Also, immunophenotypically, BM‐MSCs from healthy, obese, and morbid obese donors showed high similarity (*p* > 0.05), displaying high expression of CD29, CD73, CD90, and CD105 and low/absent expression of CD45 and HLA‐DR (Figure 1).

**TABLE 1:** Clinically relevant data of bone marrow donors.

Group	Age (years)	BMI (kg/m^2^)
Healthy	26.3 ± 8.5	21.5 ± 0.5
Obese	25.0 ± 6.2	28.7 ± 1.2*
Morbid obese	34.3 ± 4.5	34.6 ± 5.1*

* *p* < 0.05 in comparison to the healthy donor group.

### 3.2 Increasing BMI is Correlated With Loss of Stem Cell Functions in BM‐MSCs

**FIGURE 2:**
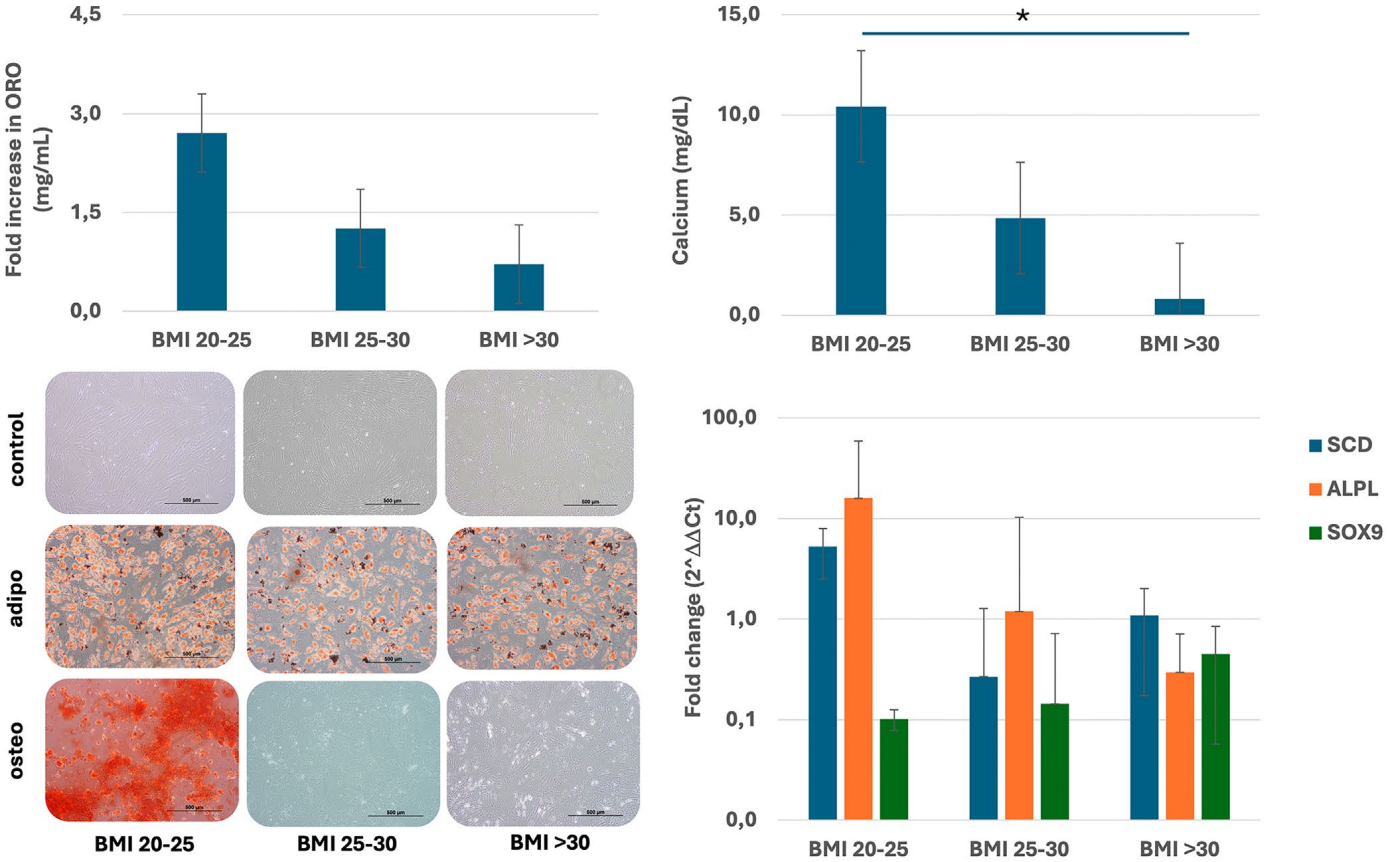
Increased BMI affects adipogenic, osteogenic and chondrogenic differentiation. BM‐MSCs from healthy (BMI 20–25), obese (BMI 25–30) and morbid obese (BMI > 30) donors were differentiated towards adipogenic, osteogenic and chondrogenic lineage. Upper left: Fold increase in ORO dye content (mg/mL) in comparison to controls after 3 weeks of adipogenic differentiation; Upper right: Calcium content (mg/dL) in wells after 3 weeks of osteogenic differentiation; Lower left: Before and after 3 weeks of adipogenic differentiation (ORO) and osteogenic differentiation (ARS); Lower right: RT‐qPCR results of early differentiation markers of adipogenic differentiation (*SCD*, blue), osteogenic differentiation (*ALPL*, orange) and chondrogenic differentiation (*SOX9*, green) after 1 week of differentiation.

Differentiation capacity of BM‐MSCs towards adipogenic, osteogenic and chondrogenic lineage decreased with increasing BMI (Figure 2), as apparent from a decrease in ORO staining and calcium phosphate depositions (*p* < 0.05) at 3 weeks of differentiation, respectively. In addition, expression of early adipogenic, osteogenic and chondrogenic differentiation‐related genes assessed at 1 week after initiation of differentiation assays revealed normal differentiation towards adipogenic (*SCD*) and osteogenic (*ALPL*) by healthy donor BM‐MSCs, in comparison to decreased differentiation of obese and morbid obese donor‐derived BM‐MSCs and overall low expression of the chondrogenic differentiation marker *SOX9*.

### 3.3 Increasing BMI is Correlated With Increased Cellular Stress in BM‐MSCs

**FIGURE 3:**
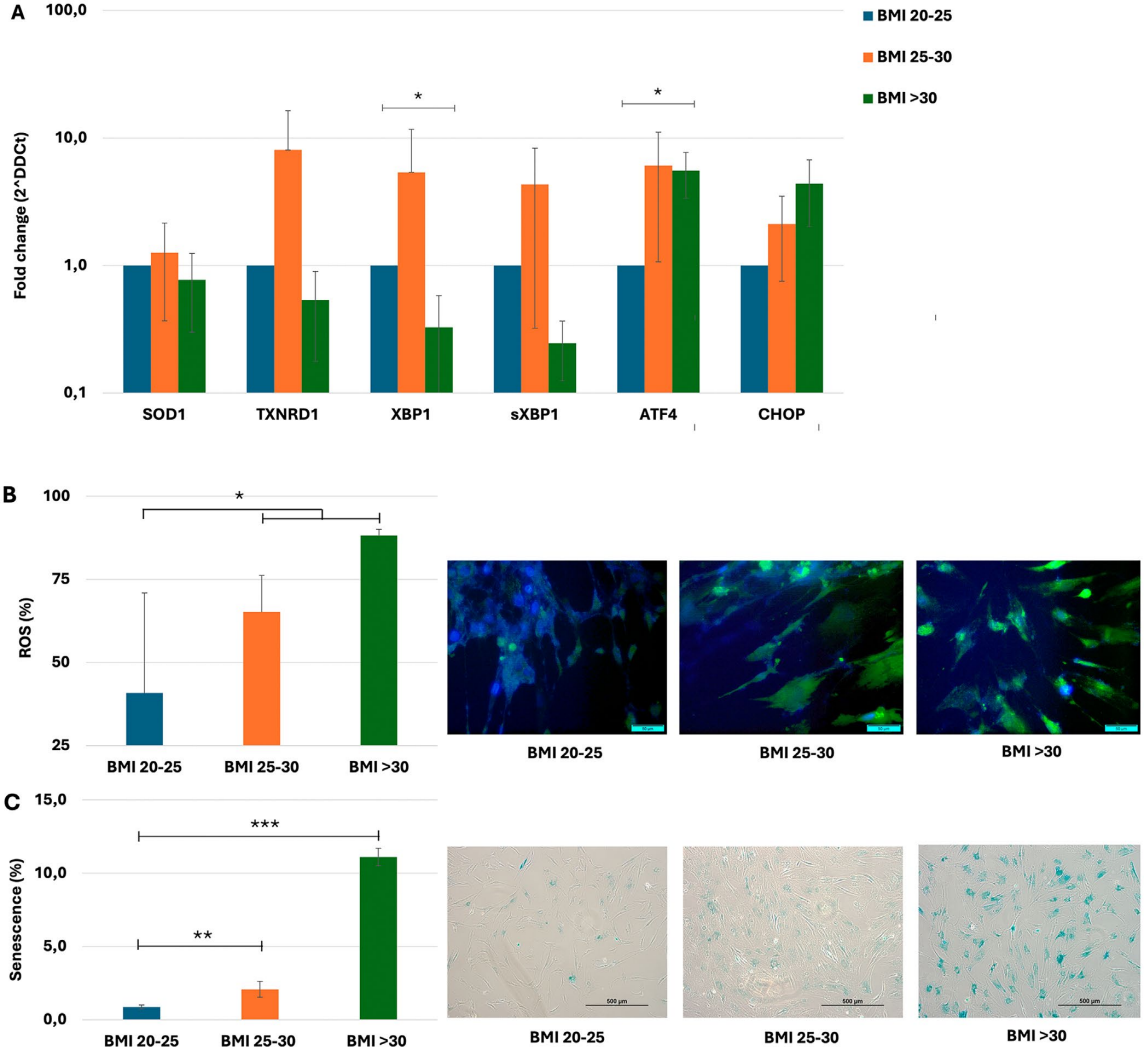
BMI‐related cellular stress in BM‐MSCs. BM‐MSCs from healthy (BMI 20–25, blue), obese (BMI 25–30, orange) and morbid obese (BMI > 30, green) donors were assessed for cellular stress markers, (*n* = 3 per group). (A) RT‐qPCR analysis of oxidative markers *SOD1* and *TRXNRD1* and ER stress markers *XBP1/sXBP1, ATF4* and *CHOP*; (B) ROS expression (%) by flow cytometry (left) and immune fluorescence microscopy (right) for H2DCFDA; (C) Percentage of senescent cells stratified per group (left) and histological staining for SA‐β‐Gal as a marker of senescence. Given are mean ± SD. *p < 0.05, **p < 0.01, ***p < 0.001.

To assess baseline levels of cellular stress in BM‐MSCs from healthy, obese and morbid obese donors, the expression of exclusively OS‐related *SOD1* and *TRXNRD1* genes and both OS and ER stress‐related genes *XBP1/sXBP1*, *ATF4* and *CHOP* was measured. Although an eight‐fold increase in *TRXNRD1* was observed in the BMI 25–30 group, these differences were not significant. However, the five‐fold increase in *ATF4* in the BMI > 30 group was significant (*p* < 0.05), as was the relative decrease of *XBP1* in the same group (*p* < 0.05) (Figure 3A). However, the ratio between XBP1/sXBP1 (0.70) remained largely unchanged between the groups. In addition, both ROS (Figure 3B) and senescent cells (Figure 3C, *p* < 0.01) accumulated notably with increasing BMI, indicating not only the presence of ongoing cellular stress, but also a relationship with increasing body weight. To confirm that cells defined as ‘senescent’ were no longer proliferatively active, cells from one donor of each group were stained with Ki67. Whereas Ki67+/SA‐β‐Gal‐ cells were found in the healthy donors (BMI < 25), Ki67 staining was absent from SA‐β‐Gal+ cells in BMI 25–30 and BMI > 30 samples (Figure S1).

### 3.4 Cellular Stress During Differentiation of BM‐MSCs

**FIGURE 4:**
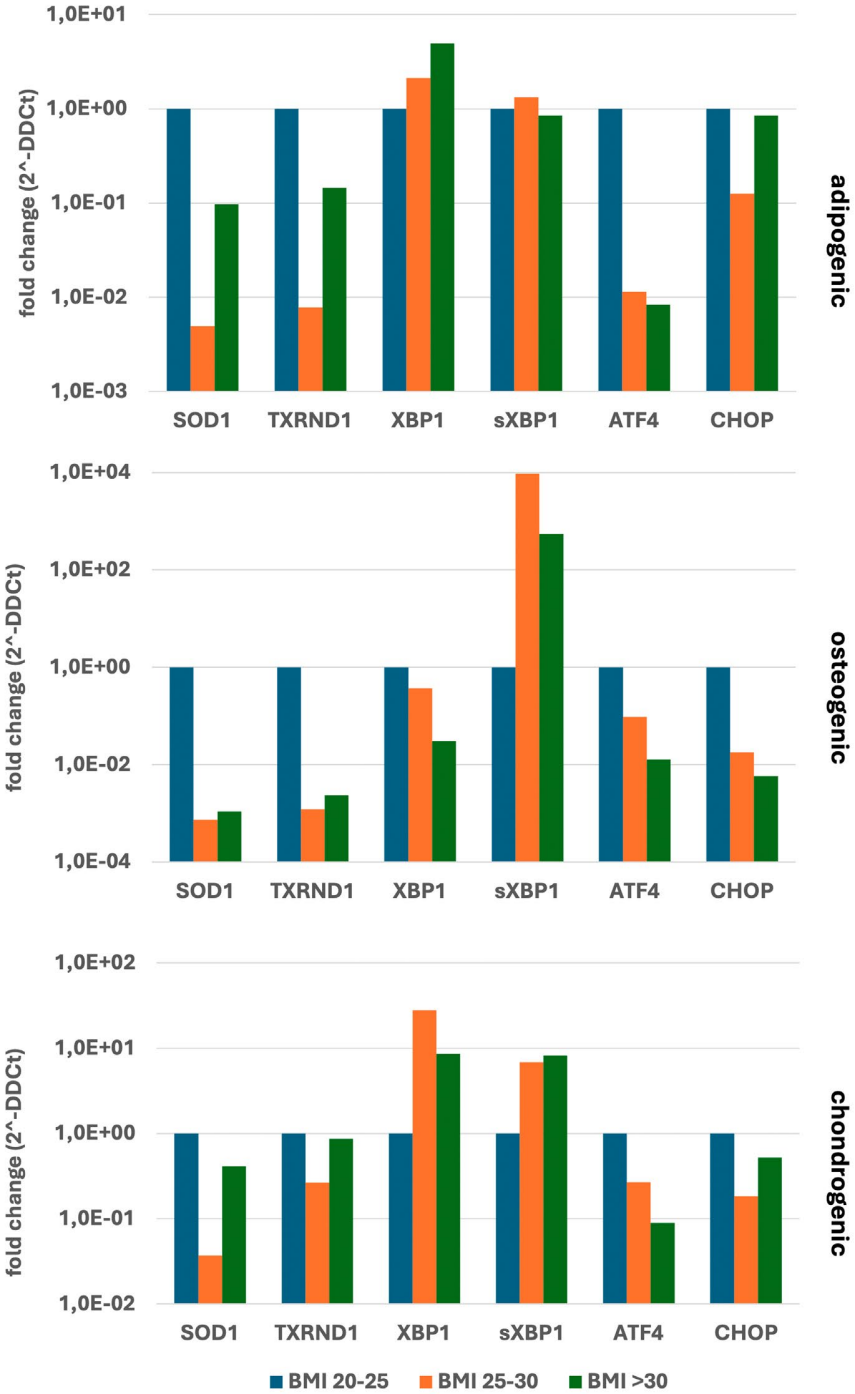
Expression of cellular stress‐related genes during differentiation of BM‐MSCs. Expression of cellular stress related genes (*SOD1*, *TXRND1*, *XBP1/sXBP1*, *ATF4* and *CHOP*) in BM‐MSCs from healthy (BMI 20–25, blue), obese (BMI 25–30, orange) and morbid obese (BMI > 30, green) donors after 1 week of differentiation in presence of adipogenic (upper panel), osteogenic (middle panel) and chondrogenic (lower panel) differentiation media. Data represent the average of *n* = 3 technical replicates.

We next wanted to assess the kinetics of cellular stress fluctuations during differentiation into adipogenic, osteogenic, and chondrogenic lineages and the relationship between cellular stress, differentiation, and increased body weight. We found that gene expression of cellular stress genes during adipogenic differentiation was only minimally affected, whereas during osteogenic differentiation the presence of *sXBP1* showed a noticeable increase (Figure 4). These data are in line with our (previous) findings that showed that although both osteogenic and adipogenic differentiation are affected by ER stress, the phenotype is more severe during osteogenic differentiation [2]. Stress during chondrogenic differentiation of obese and morbidly obese donors has not been assessed before, but our data clearly show an increase in both *XBP1* and *sXBP1* expression, indicating that obesity‐related stress conditions may also affect chondrogenic differentiation of BM‐MSCs.

### 3.5 Modulation of Cellular Stress Using TUDCA and Melatonin

The dose and working mechanism of TUDCA have been determined previously [2]. To determine the optimal dose of MT, potential interactions with TUDCA and/or toxic effects related to concentration, we performed a series of optimisation experiments (Figure S2), based on which we determined the optimal dose of MT to be 30 nM. Whereas both TUDCA and MT stimulated proliferation of healthy BM‐MSCs in comparison to standard DMF10 medium controls, co‐administration of TUDCA and MT did not result in additive or synergistic effects. Therefore, in subsequent experiments, TUDCA and MT were used separately. To assess the mechanisms through which MT exerts its effects on BM‐MSCs, we also studied downstream activation of pERK and pAKT (Figure S3). We found that MT induction resulted in rapid downstream phosphorylation of ERK, but not AKT, indicating that MT actions are largely mediated through activation of the MT2 receptor on the cell surface of BM‐MSCs.

**FIGURE 5:**
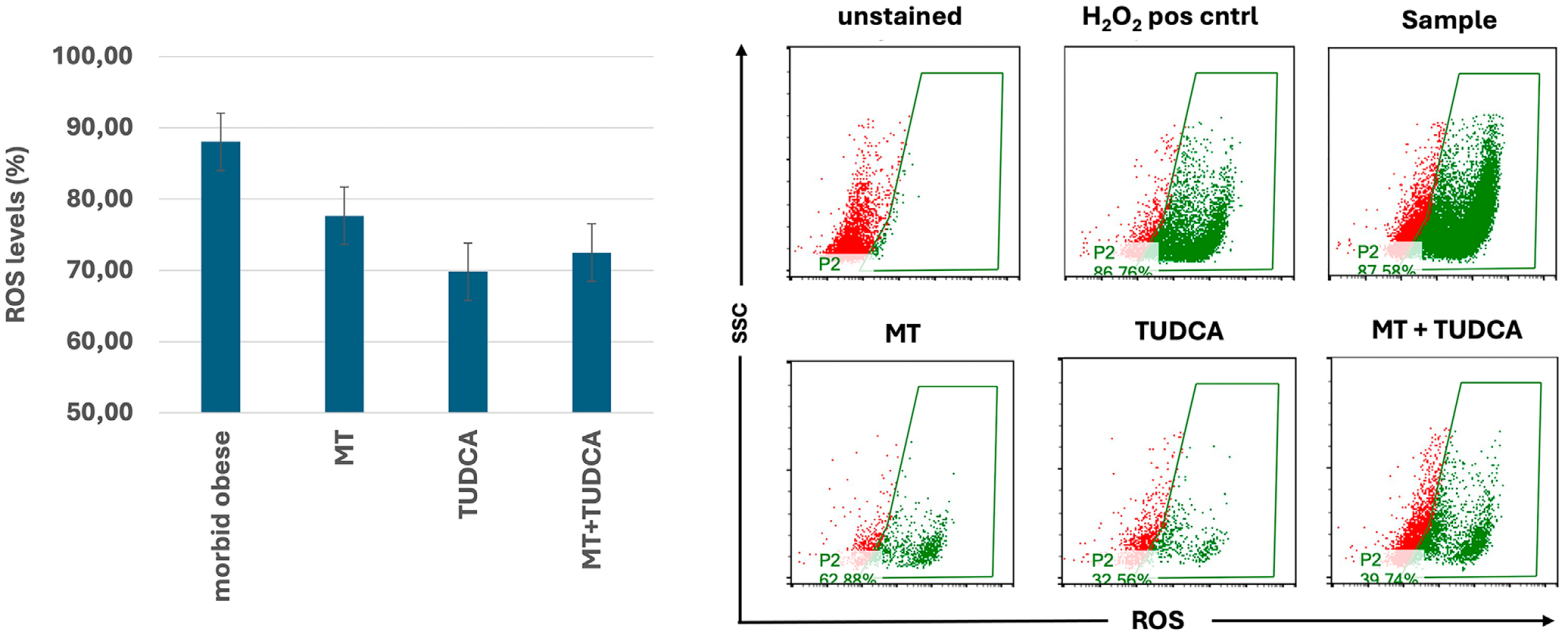
Effect of MT and TUDCA on ROS levels in high BMI (> 30) BM‐MSCs. ROS levels (%) in morbid obese donor‐derived BM‐MSCs (BMI > 30) were measured using a H2DCFDA kit before and after treatment with MT (Melatonin), TUDCA or both. Left: Average ± standard deviation of *n* = 3 morbid obese donors. Right: Representative FACS dot plots measuring green fluorescence in presence of ROS. H_2_O_2_ was used as a positive control.

**FIGURE 6:**
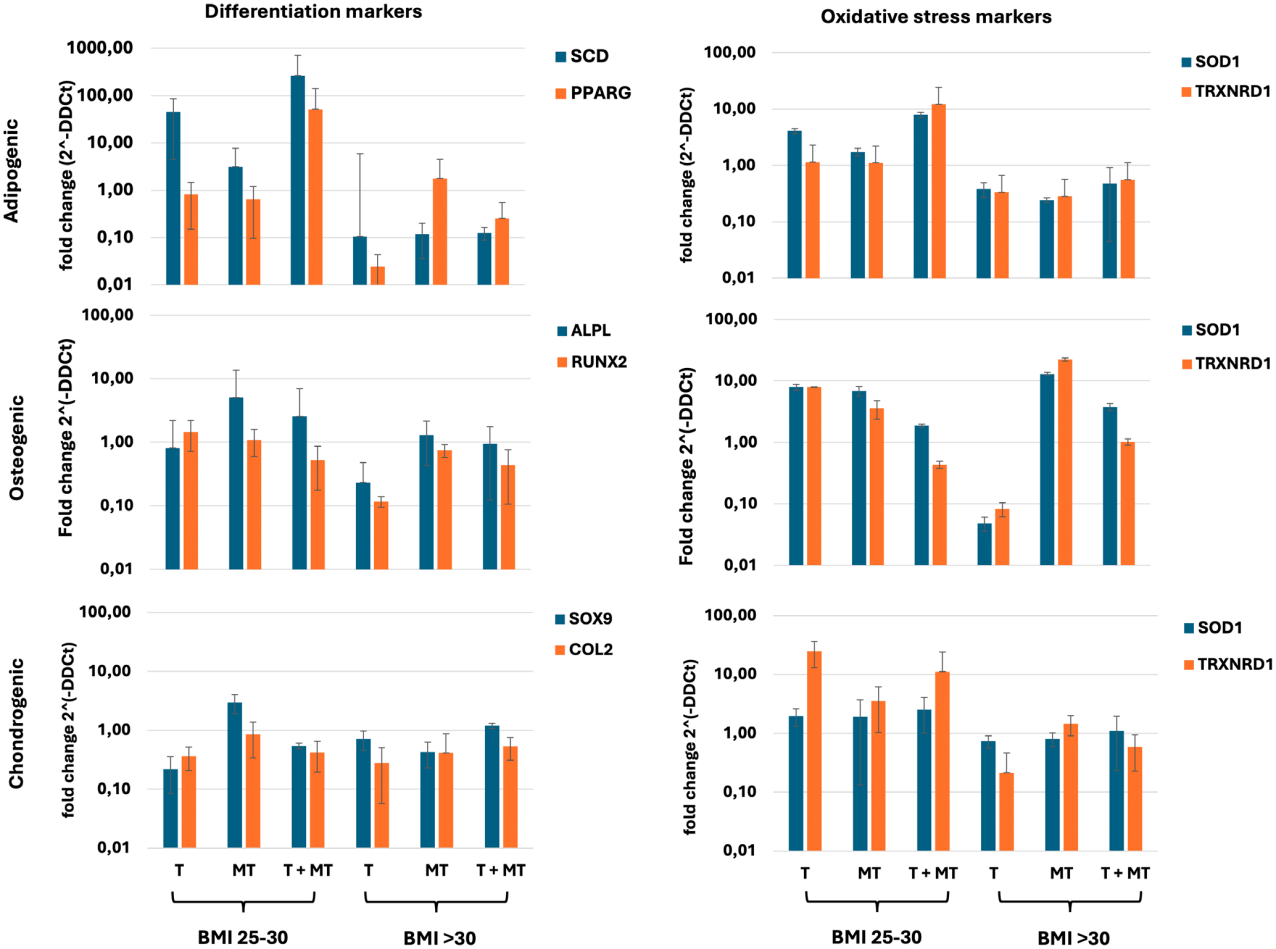
Effect of MT and TUDCA on differentiation and oxidative stress in obese and morbid obese donor BM‐MSCs. BM‐MSCs from obese (BMI 25–30) and morbid obese (BMI > 30) donors were subjected to 1 week of differentiation towards adipogenic (upper panel), osteogenic (middle panel) and chondrogenic (lower panel) lineage. Expression of lineage‐specific differentiation markers (left) and markers of oxidative stress (right) were assessed in presence of TUDCA (T), Melatonin (MT) or both (T + MT). Data were compared to differentiation and oxidative stress levels in the same sample in the absence of T or MT and represent the average of *n* = 3 technical replicates.

We then assessed the effects of MT and TUDCA on ROS levels and OS in BM‐MSCs from obese and morbid obese donors. Morbid obese donor BM‐MSCs were treated with MT, TUDCA, or a combination thereof (Figure 5). Although ROS levels remained high in all samples, on average, a 10%–15% decrease in ROS levels could be observed in any treatment group (*p* > 0.05). Next, we monitored the differentiation capacity of these BM‐MSCs in the presence of MT and TUDCA (Figure 6). Modulation of OS using MT, TUDCA, or a combination thereof did only marginally affect the differentiation potential of the cells, with a small increase in *SCD*, *ALPL*, and *SOX9* expression in obese donor BM‐MSCs during adipogenic, osteogenic, and chondrogenic differentiation, respectively, but did not correct or improve the differentiation of BM‐MSCs from morbid obese donors. Similarly, although an increase in gene expression of the oxidative enzymes *SOD1* and *TRXNRD1* was observed in the obese donor BM‐MSC group, in the morbid obese donor BM‐MSC group, expression was almost always lower, with the exception of *SOD1* and *TRXNRD1* during osteogenic differentiation after treatment with MT. These data indicate that despite ongoing OS, compensatory mechanisms induced to cope with cellular stress are failing in BM‐MSCs of morbid obese individuals, resulting in a lack of response to MT and TUDCA.

**FIGURE 7:**
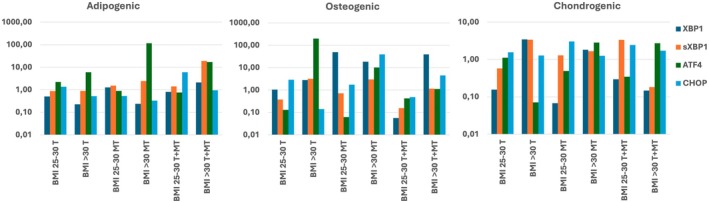
Effect of MT and TUDCA on markers of ER stress in obese and morbid obese donor BM‐MSCs. BM‐MSCs from obese (BMI 25–30) and morbid obese (BMI > 30) donors were subjected to 1 week of differentiation towards adipogenic (left), osteogenic (middle) and chondrogenic (right) lineage. Expression of markers of endoplasmic reticulum stress were assessed in presence of TUDCA (T), Melatonin (MT) or both (T + MT). Data were compared to differentiation and ER stress levels in the same sample in the absence of T or MT and represent the average of *n* = 3 technical replicates.

We previously found that ER stress is a contributing factor to the loss of stemness and differentiation potential in obese donor BM‐MSCs [2]. However, treatment with TUDCA could, at least partially, restore osteogenic and adipogenic differentiation potential. Here, we focused on differences between obese and morbid obese donor‐derived BM‐MSCs (Figure 7). We found that suppression of the expression of *XBP1* and *sXBP1* by TUDCA resulted in somewhat improved adipogenic differentiation, but was hardly or not effective in improving osteogenic or chondrogenic differentiation. Also, treatment with TUDCA was only effective in the obese donor‐derived BM‐MSCs, and showed no improvement in differentiation or a decrease of ER stress markers in morbid obese BM‐MSCs. Treatment with MT did neither improve differentiation capacity of obese and morbid obese donor BM‐MSCs nor did it increase expression of OS‐related antioxidant enzymes. However, we observed overall increased expression of the transcription factor *ATF4*, which is linked to both OS and ES, in BM‐MSCs from morbid obese donors. Simultaneous treatment of cells with both TUDCA and MT increased the stress response to OS, improved adipogenic differentiation, but not osteogenic and chondrogenic differentiation. No consistent effects were observed in suppression of ER stress markers.

## 4 Discussion

Obesity has become a global public health problem and increases the likelihood of many metabolic diseases. It has been shown in multiple studies that obesity may affect stem cell function negatively [2, 3, 4, 5]. Here, we aimed to elucidate the mechanisms involved in the loss of stem cell function induced by obesity by assessing levels of oxidative stress (OS) and endoplasmic reticulum stress (ERS) in bone marrow‐derived mesenchymal stromal cells (BM‐MSCs) from otherwise healthy donors with a BMI of 25–30 (obese) and BMI > 30 (morbid obese). We found that although BMI was significantly different between the groups, differences in donor age were not significant. Although we found a trend with overall BMI increasing with age, there was a significant positive correlation between age and BMI in the selected donors. However, even under these circumstances, it cannot be concluded that age does not affect any of the variables tested in this study. Indeed, when some of our results were re‐analysed for correlation with age, we found a trend for an increase in variables such as senescence and ER stress‐related gene expression levels with advancing age (not significant). In addition, cellular immunophenotype and ROS levels and OS‐related gene expression did not show any (positive) correlation with increasing age (Figure S4). To completely rule out any involvement of age as a confounding factor, however, the sample size of the study groups should be increased to further differentiate between a specific BMI effect and an age‐related effect.

We compared the basic levels of OS and ERS, as well as the induction of cellular protection mechanisms activated by cellular stress, including increases in *SOD1* and *TRXNRD1* expression in response to OS and induction of the UPR, by assessing *XBP1/sXBP1*, *ATF4*, and *CHOP* expression. We did not confirm our gene expression data with assessment of activation of UPR or oxidative stress at the protein level. One reason was that we used functional assays rather than western blot to confirm loss of differentiation potential, as we did before [2]. Another was that we used measurement of ROS levels as confirmation of OS. However, future studies are warranted to further explore the direct effects of BMI on ERS and OS at a protein level and to validate the differences in stress‐related gene expression as reliable stress proxies. We have previously shown that obesity results in increased ER stress in BM‐MSCs and loss of stemness, which can be partially recovered by decreasing ERS with TUDCA [2]. However, we did not at that point assess the relationship between the degree of obesity and BM‐MSC function, nor did we assess the involvement of other cellular stress factors, including OS. Here, we found morphologically or immunophenotypically no differences between BM‐MSCs from healthy, obese, and morbid obese donors, indicating that morphological assessment and basic immunophenotyping [12] is not sufficient to directly identify affected cells. However, BM‐MSC three‐lineage differentiation potential into adipogenic, osteogenic, and chondrogenic was directly related to the degree of obesity and resulted in increased levels of intracellular ROS, increased senescence‐associated β‐Galactosidase expression, upregulation of OS and ERS‐related genes, and induction of cellular protection mechanisms, as evident from increased expression of *TRXNRD1*, *XBP1/sXBP1*, *ATF4*, and *CHOP*.

We then assessed the effect of the antioxidant Melatonin (MT), which is known to decrease OS and protect tissues from ischemia/reperfusion injury [23] and TUDCA, a bile acid with chaperoning activity [29], known to decrease ERS by preventing activation of the UPR. In humans, MT has four different receptors, of which three are membrane receptors and one functions as a nuclear receptor for MT. Due to its high lipid solubility, MT can, independently from interactions with its receptors, freely diffuse through the cell membrane and serve as a potent oxygen radical scavenger [23]. Although the presence of MT receptors has not been directly confirmed on BM‐MSCs, the use of MT antagonists indicates that signalling in MSCs occurs through activation of MT2 receptors [30]. Although MT receptor expression studies in BM‐MSCs are lacking, in ADSCs, expression of both MT1 and MT2 receptors has been confirmed [31]. In order to assess which receptor is involved in signal transduction of MT in BM‐MSCs, we measured phosphorylation of ERK and AKT pathways. It has been previously shown that activation of MT1 signalling results in the activation of the PI3K pathway, with downstream phosphorylation and activation of both pAKT and pERK1/2 [32, 33]. In contrast, activation of the MT2 signalling pathways is mediated through activation of PKC and downstream phosphorylation of pERK1/2, but not activation of pAKT. In our experiments, we found only increased phosphorylation of pERK, but not pAKT, indicating that the effects of MT on BM‐MSCs are mainly mediated through activation of MT2, which is in line with previously published literature [30]. TUDCA is a bile acid conjugated to taurine that has been shown to possess potent chemical chaperone activity [34, 35], through which it can inhibit both ERS and decrease OS via suppression of ROS production, thus providing a protective mechanism in high glucose diet‐induced obesity models. TUDCA mainly works through activation of its three membrane receptors, the G protein‐coupled bile acid receptor (TGR5), Sphingosine‐1‐phosphate receptor 2 (S1PR2), andα5β1 integrin, and its nuclear receptor Farnesoid X receptor (FXR) [36, 37]. However, some actions of TUDCA have also been shown to be mediated through interactions with the mineralocorticoid receptor and glucocorticoid receptor [38].

We hypothesised that since the mode of action of MT and TUDCA (different receptors and activation pathways), as well as their main targets (OS and ERS, respectively) are overlapping but still largely distinct, these chemicals may have a synergistic effect when used together to suppress cellular stress in obesity‐affected stem cells. However, although treatment with either MT or TUDCA resulted in a decrease in intracellular ROS levels, combined treatment with both agents did not result in a further decrease. When we applied MT, TUDCA or both during differentiation of BM‐MSCs from obese and morbid obese donors we found that especially during adipogenic and osteogenic differentiation, suppression of OS and ERS resulted in almost complete recovery of differentiation of BM‐MSCs from obese donors, in comparison to healthy controls. However, treatment of BM‐MSCs from morbid obese donors with MT, TUDCA or both was never sufficient to recover loss of differentiation potential, indicating that loss of differentiation potential in BM‐MSCs from obese donors can be partially counteracted, but loss of differentiation potential in BM‐MSCs from morbid obese donors appears to be irreversible. Chondrogenic differentiation of both BM‐MSCs from obese and morbid obese donors was similarly decreased but neither treatment with TUDCA nor with MT or a combination thereof was sufficient to improve chondrogenic gene expression. Interestingly, in the morbid obese group *SOD1* and *TRXNRD1* expression levels returned to ‘normal’ levels in the absence of chondrogenic differentiation, indicating that the mechanisms designed to cope with increased OS in obese individuals are failing.

A recent study in both mice and humans revealed the presence of obesity‐induced epigenetic changes in the genome of adipocytes, causing persistent transcriptional deregulation even after weight loss [39]. In addition, it has been shown on multiple occasions that obesity affects tissue‐specific stem cell function. One underlying mechanism of this long‐term effect on stem cell function may be the activation and induction of (irreversible) changes in homeostatic mechanisms regulating energy balance [40]. Another may be related to ongoing cellular stress, senescence, and inflammation. Our current results are in line with these studies.

Based on these data, we believe that activation of cellular stress in BM‐MSCs from morbidly obese donors (BMI > 30) was irreversible and may initially cause individual cells to lose their stemness potential, but may eventually result in depletion of the stem cell pools. These data indicate that it is imperative to treat and prevent obesity before the negative effects on stem cells become permanent and irreversible. Treatment of obesity at an early stage may not only result in a decrease in metabolic diseases, it may also support overall tissue health by supporting the tissue regeneration capacity of the resident stem cells.

## Author Contributions

**Ece Gizem Polat:** conceptualization (equal), data curation (equal), formal analysis (equal), investigation (equal), methodology (equal), writing – original draft (equal). **Mehmet Emin Şeker:** formal analysis (equal), investigation (equal), methodology (equal). **Burcu Pervin:** methodology (equal). **Barış Ulum:** data curation (supporting), investigation (supporting). **Fatima Aerts‐Kaya:** conceptualization (equal), data curation (equal), formal analysis (equal), funding acquisition (equal), investigation (equal), methodology (equal), project administration (equal), resources (equal), supervision (equal), writing – review and editing (equal).

## Ethics Statement

This study was approved by the Ethics Committee Hacettepe University (GO:2022/11‐68).

## Conflicts of Interest

The authors declare no conflicts of interest.

## Supporting information


**Data S1:** jcmm70776‐sup‐0001‐Supinfo.docx.

## Data Availability

The data that support the findings of this study are available from the corresponding author upon reasonable request.
