# Supplementary material for: Obesity‐Induced Loss of Function of Bone Marrow Mesenchymal Stromal Cells Is Linked to Cellular Stress and Irreversible at Advanced Stages
Source: J Cell Mol Med. 2025 Aug 7;29(15):e70776. doi: 10.1111/jcmm.70776 (PMC12331444; doi:10.1111/jcmm.70776)
Supplement: Supplementary file 1 — Data S1: jcmm70776‐sup‐0001‐Supinfo.docx. [file JCMM-29-e70776-s001.docx]

**Obesity-induced loss of function of bone marrow mesenchymal stromal cells is linked to cellular stress and irreversible at advanced stages – supporting information**

Ece Gizem Polat, Mehmet Emin Şeker, Burcu Pervin, Barış Ulum, Fatima Aerts-Kaya

**Figure S1 Ki67 staining of senescent cells**

**
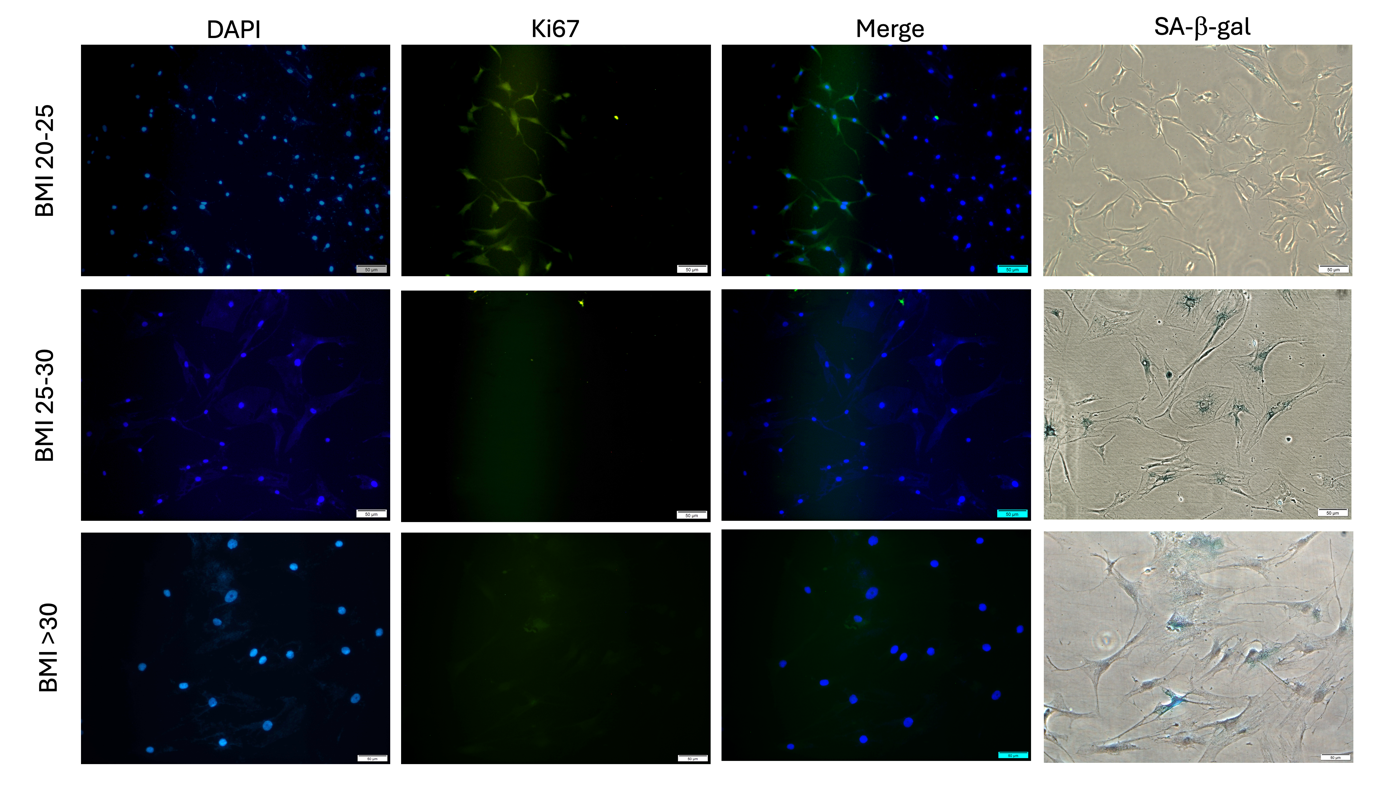
**

BM-MSCs from a healthy donor (BMI 20-25), an obese donor (BMI 25-30) and a morbid obese donor (BMI >30) were stained with senescence-associated-β-Galactosidase (blue, light microscope) and counterstained with the proliferation marker Ki67 (green fluorescent) and DAPI (blue fluorescent). Ki67+ cells were only observed in the samples from healthy donors, but not obese donors. Thus, increasing weight was associated with an increased presence of non-proliferative Ki67-/SA-β-Gal+ senescent cells.

**Figure S2 Effect of MT and TUDCA on proliferation of healthy donor BM-MSCs**

**
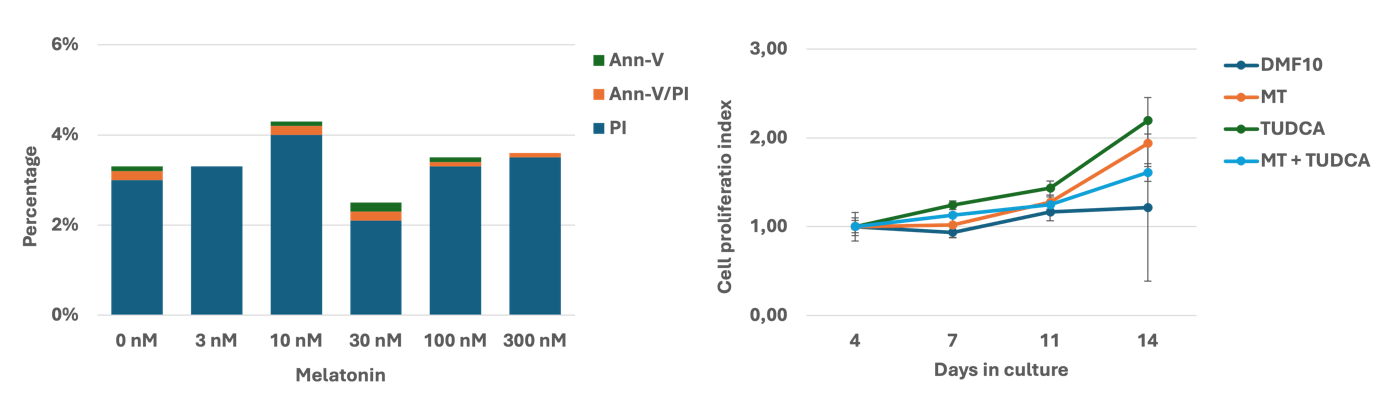
**

BM-MSCs from a healthy donor were used to determine the optimal dose of MT in culture. Left: Doses of 3 nM-300 nM MT were added to the culture medium of BM-MSCs and 24 hours apoptosis was assessed using Annexin-V/Propidium Iodide staining (Ann-V/PI). Although we did not observe any important toxicity, we decided to use a dose of 30 nM MT based on these data. Right: Use of MT and/or TUDCA during prolonged culture of BM-MSCs (up to 14 days) showed that both MT and TUDCA independently supported proliferation, whereas co-stimulation with both agents resulted in an inhibitory effect. Therefore in subsequent experiments we only applied MT and TUDCA separately.

**Figure S3 MT activates MT2 on BM-MSCs and induces rapid phosphorylation of ERK**

**
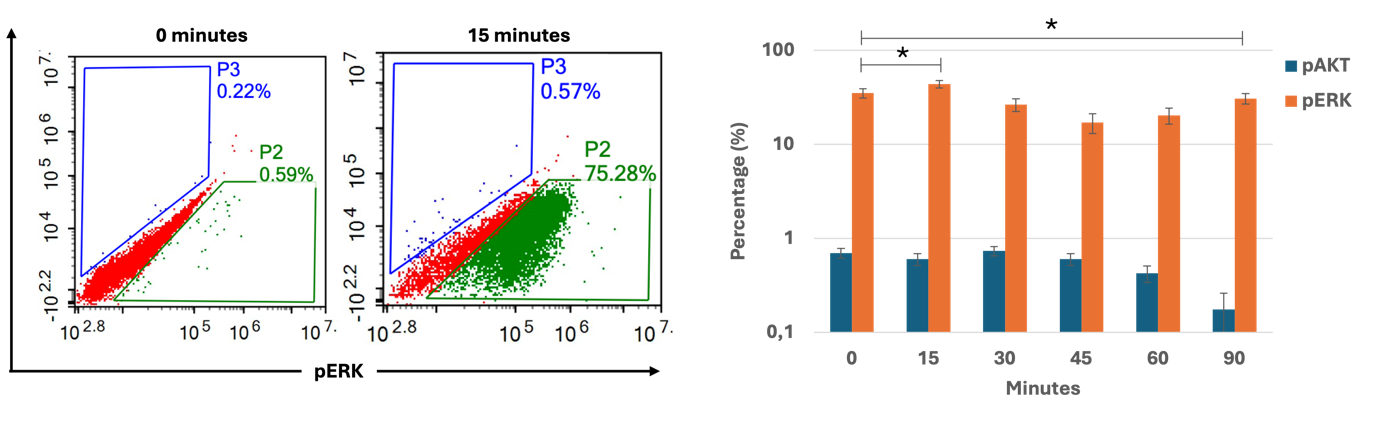
**

BM-MSCs from healthy BM donors were cultured in DMF10 medium and starved for 24 hours before addition of 30 nM MT. Cells were incubated for 15, 30, 45, 60 or 90 minutes in presence of MT, after which they were fixed and stained for expression of phosphorylated (p) AKT and ERK. Although no differences were observed in expression and phosphorylation status of AKT, a rapid and reversible increase in pERK was observed, indicating that signaling of MT in BM-MSCs is largely mediated by the MT2 receptor, which is known to activate downstream signaling through phosphorylation of ERK, but not AKT.

**Figure S4 Age alone is not responsible for differences in cellular stress and senescence**

**
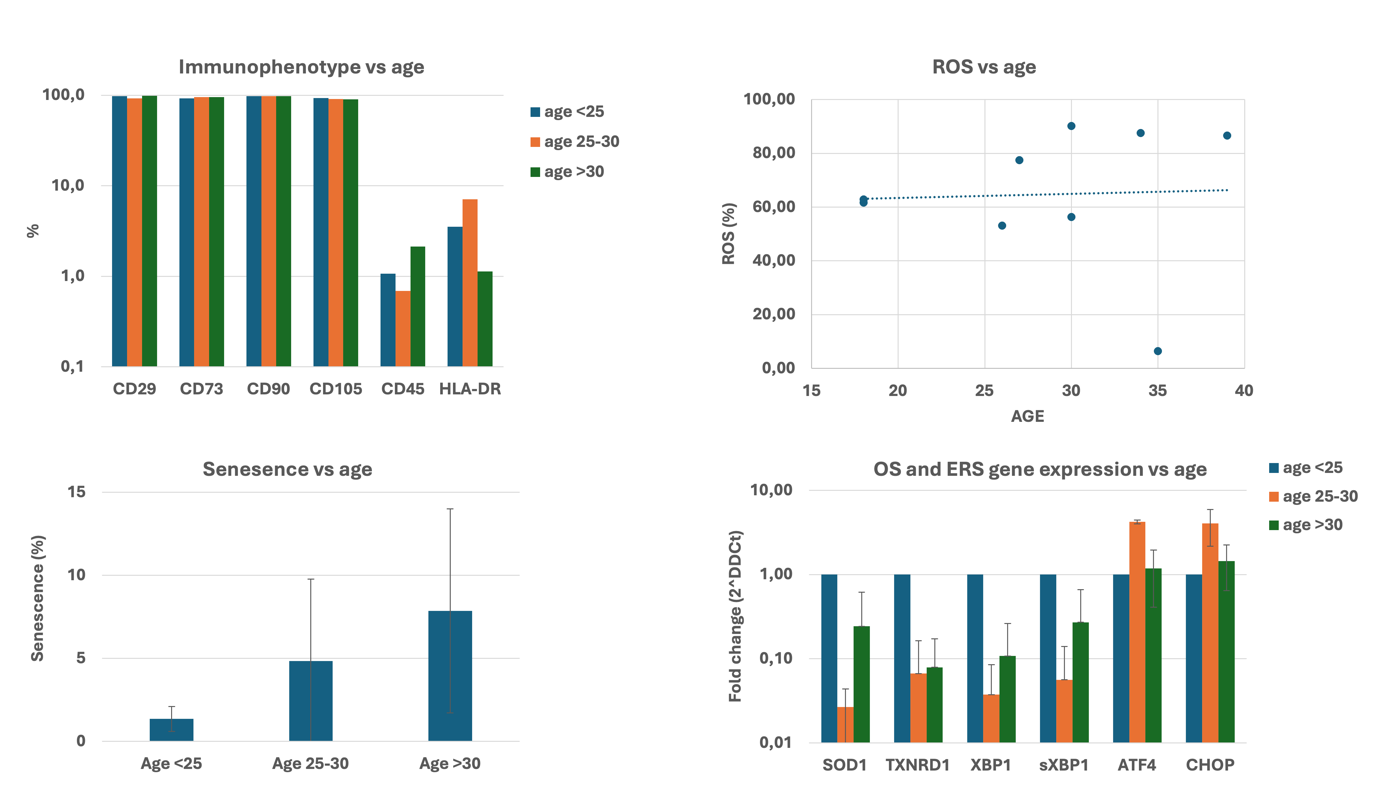
**

Data obtained using the healthy, obese and morbid obese BM-MSC donors were re-analyzed to reveal any effects on the results caused by increasing age. The donors were stratified according to age into three groups, i.e. <25 years, age 25-30 and age >30 years. No significant correlation was found between increasing age and immunophenotype, ROS levels and OS-related gene expression. A trend was observed for increased levels of senescence and increased levels of ERS-related gene expression, but these were not significant either. Therefore, we can exclude the effect of age as an important confounding factor to this study, although it may have contributed to some of the effects seen.

**Table S1. Primers used for RT-qPCR**

| **Gene** | **Forward** | **Reverse** | **Marker** |
| --- | --- | --- | --- |
| *PPARG* | CAGCACCACCGATCAGAAGA | TCCCATTTCCGAGGAGGGAT | Adipogenic differentiation |
| *SCD* | CCCCTGCTTACTTGGTGAGG | TGTTCAGCAGGGTTTGTGGT | Adipogenic differentiation |
| *RUNX2* | CGCCTCACAAACAACCACAG | TCACTGTGCTGAAGAGGCTG | Osteogenic differentiation |
| *ALPL* | GCTATCCTGGCTCCGTGC | CACTGTGGAGACACCCATCC | Osteogenic differentiation |
| *COL2* | GGTCCTGCAGGTGAACCC | CTCTGTCTCCTTGCTTGCCA | Chondrogenic differentiation |
| *SOX9* | AGACAGCCCCCTATCGACTT | CGGCAGGTACTGGTCAAACT | Chondrogenic differentiation |
| *sXBP1* | GCTGAGTCCGCAGCAGGT | CTGGGTCCAAGTTGTCCAGAAT | ER stress/UPR |
| *XBP1* | CAGACTACGTGCACCTCTGC | CTGGGTCCAAGTTGTCCAGAAT | ER stress/UPR |
| *CHOP* | GCTCAGGAGGAAGAGGAGGA | TCCTGCTTGAGCCGTTCATT | ER stress/UPR |
| *ATF4* | TTAAGCCATGGCGCTTCTCA | GGTCGAAGGGGGACATCAAG | ER stress/UPR |
| *SOD1* | CTCACTCTCAGGAGACCATTGC | CCACAAGCCAAACGACTTCCAG | Oxidative stress |
| *TXNRD1* | GTTACTTGGGCATCCCTGGTGA | CGCACTCCAAAGCGACATAGGA | Oxidative stress |
| *RPLP0* | TGGTCATCCAGCAGGTGTTCGA  ACAGACACTGGCAACATTGCGG | ACAGACACTGGCAACATTGCGG | Housekeeping gene |
